# Generalized matrix summability of a conjugate derived Fourier series

**DOI:** 10.1186/s13660-017-1542-5

**Published:** 2017-11-02

**Authors:** M Mursaleen, Abdullah Alotaibi

**Affiliations:** 1 Department of Mathematics, Aligarh Muslim University, Aligarh, 202002 India; 2 Operator Theory and Applications Research Group, Department of Mathematics, Faculty of Science, King Abdulaziz University, P.O. Box 80203, Jeddah, 21589 Saudi Arabia

**Keywords:** 42A24, 40G05, Fourier series, conjugate Fourier series, conjugate derived Fourier series, $(E,q)$-summability, $(E,q)(\bar{N},p_{n})$-summability, $F_{\mathfrak{B}}$-convergence

## Abstract

The study of infinite matrices is important in the theory of summability and in approximation. In particular, Toeplitz matrices or regular matrices and almost regular matrices have been very useful in this context. In this paper, we propose to use a more general matrix method to obtain necessary and sufficient conditions to sum the conjugate derived Fourier series.

## Introduction

The idea of $\mathfrak{B}$-summability (or $F_{\mathfrak{B}}$-convergence) was introduced by Bell [1] and Steiglitz [2]. It generalizes the notions of *A*-summability and almost convergence.

Let $\mathfrak{B}=(B_{i})_{i=1}^{\infty}$ be a sequence of infinite matrices with $B_{i}=(b_{nk}(i))_{n,k=1}^{\infty}$. Then a bounded sequence $x=(x_{k})_{i=1}^{\infty}$ is said to be $\mathfrak{B}$-summable (or $F_{\mathfrak{B}}$-convergent) to the value *L* if $\lim_{n}(B_{i}x)_{n}=\lim_{n}\sum_{k}b_{nk}(i)x_{k}=L$, uniformly in $i\geq 0$. In this case, *L* is denoted $\mathfrak{B}$-lim*x*.

Note that for $\mathfrak{B}=I$ (the unit matrix), $F_{\mathfrak{B}}$-convergence is reduced to the ordinary convergence of *x*. For $\mathfrak{B}=\mathfrak{B}_{1}$, then $F_{\mathfrak{B}}$-convergence is reduced to the almost convergence of *x* (see Lorentz [3]), where $\mathfrak{B}_{1} =(b_{nk}^{1}(i))_{n,k=1}^{\infty}$ with

$$ b_{nk}^{1}(i)= \textstyle\begin{cases} \frac{1}{n+1}, & n\leq i\leq n+k, \\ 0 & \text{otherwise.}\end{cases} $$

In this paper, we use such type of matrices, which have many applications in various fields, to study the summability problem of the conjugate derived Fourier series.

Let $f(t)$ be a periodic function with period 2*π* and Lebesgue-integrable over $[-\pi,\pi]$. Then the Fourier series associated with *f* at any point *x* is defined by

1.1 $$ f(x)\sim\frac{a_{0}}{2}+\sum_{j=1}^{\infty} ( a_{j}\cos jx+b_{j}\sin jx ), $$

and the conjugate series of the Fourier series (1.1) is

1.2 $$ \sum_{j=1}^{\infty} ( b_{j}\cos jx-a_{j}\sin jx ). $$

The derived Fourier series is

1.3 $$ \sum_{j=1}^{\infty}j ( b_{j}\cos jx-a_{j}\sin jx ), $$

and the conjugate derived Fourier series (omitting the minus sign) is

1.4 $$ \sum_{j=1}^{\infty}j ( a_{j}\cos jx+b_{j}\sin jx ). $$

In this paper, we apply the notion of $F_{\mathfrak{B}}$-convergence to study the summability problems of the conjugate derived Fourier series (1.4).

## Preliminaries

For an infinite matrix $A=(a_{nk})_{n;k=1}^{\infty}$ of real or complex numbers, the *A*-transform of the sequence $x=(x_{k})_{k=1}^{\infty}$ is defined by the sequence $Ax=(A_{n}(x))$, provided that $A_{n}(x)=\sum_{k}a_{nk}x_{k}$ converges for each $n\in\mathbb{N}$. If $x=(x_{k})\in X$ implies that $Ax\in Y$, then we say that *A* defines a matrix transformation from a sequence space *X* into another sequence space *Y*, and we denote the class of such matrices by $(X,Y)$. Let *c* denote the space of all convergent sequences. We say that *A* is a conservative matrix if it transforms a convergent sequence into a convergent one. If, in addition, $\lim Ax=\lim x$, then *A* is called a regular matrix, and in this case, we write $A\in(c,c)_{\mathrm {reg}}$. The well-known Silverman-Töeplitz conditions for the regularity of *A* are (see [4]):

(i) $\Vert A \Vert =\sup_{n}\sum_{k}\mid a_{nk}\mid<\infty$,
(ii) $\lim_{n}a_{nk}=0$ for each *k*,
(iii) $\lim_{n}\sum_{k}b_{nk}=1$.

These regular matrices play a very important role in summing nonconvergent or convergent sequences and series. Our methods are based on the previous techniques, that is, we put conditions on the entries of a matrix through which we try to sum certain sequences or series. There are special well-known regular summability matrices, Cesàro, Riesz, Nörlund, Euler, and Hankel matrices, which have various applications in different fields of mathematics and other sciences.

### Theorem A

*The method* $\mathfrak{B}$ *is regular* (*see* [1, 2]) *if and only if*

(i) $\Vert\mathfrak{B}\Vert<\infty$,
(ii) $\lim_{n}b_{nk}(i)=0$ *for all* $k\geq1$ *uniformly in* *i*, *and*
(iii) $\lim_{n}\sum_{k}b_{nk}(i)=1$ *uniformly in* *i*,

*where*

$$ \Vert\mathfrak{B}\Vert=\sup_{n,i}\sum _{k} \bigl\vert b_{nk}(i) \bigr\vert < \infty, $$

*which means that there exists a constant* *M* *such that*

$$ \sum_{k} \bigl\vert b_{nk}(i) \bigr\vert \leq M~ $$

*for all* $n,i$ *and the series* $\sum_{k} \vert b_{nk}(i) \vert $ *converges uniformly in* *i* *for each* *n*.

*Let* $\sum_{n}a_{n}$ *be a given infinite series with the sequence of partial sums* $(s_{n})$. *Let* $(p_{n})$ *be a sequence of positive real numbers such that*

$$ P_{n}=\sum_{\upsilon=0}^{n}p_{\upsilon} \rightarrow\infty,\quad n\rightarrow\infty\ (P_{-j}=p_{-j}=0, \text{ }j\geq0). $$

*We write*

$$ t_{n}=\frac{1}{P_{n}}\sum_{\upsilon=0}^{n}p_{\upsilon}s_{\upsilon}, $$

*which denotes the* $(\bar{N},p_{n})$-*mean of the sequence* $(s_{n})$ *generated by the sequence of coefficients* $(p_{n})$. *The series* $\sum_{n}a_{n}$ *is said to be* $(\bar{N},p_{n})$-*summable to* *l* *if* $t_{n}\rightarrow l$ *as* $n\rightarrow\infty$ (*see* [5]). *The* $(\bar{N},p_{n})$-*summability method is regular if* (i) $P_{n}\rightarrow\infty$ *as* $n\rightarrow \infty$ *and* (ii) $\sum_{\upsilon=0}^{n}p_{\upsilon}\leq C\mid P_{n}\mid$ *as* $n\rightarrow\infty$.

*We write*

$$ T_{n}=\frac{1}{(1+q)^{n}}\sum_{\upsilon=0}^{n} \binom{n}{\upsilon}q^{n-\upsilon}s_{\upsilon}, $$

*which denotes the* $(E,q)$-*mean of the sequence* $(s_{n})$. *The series* $\sum_{n}a_{n}$ *is said to be* $(E,q)$-*summable to* *l* *if* $T_{n}\rightarrow l$ *as* $n\rightarrow\infty$ (*see* [5]). *The* $(E,q)$-*summability method is regular*.

*Further*, *we write*

$$ \tau_{n}=\frac{1}{(1+q)^{n}}\sum_{k=0}^{n} \binom{n}{k}q^{n-k}\frac {1}{P_{k}}\sum _{\upsilon=0}^{k}p_{\upsilon}s_{\upsilon}, $$

*which denotes the* $(E,q)$-*transform of the* $(\bar{N},p_{n})$-*mean of the sequence* $(s_{n})$. *The series* $\sum_{n}a_{n}$ *is said to be* $(E,q)(\bar {N},p_{n})$-*summable to* *l* *if* $\tau_{n}\rightarrow l$ *as* $n\rightarrow \infty $ (*see* [6]).

We also need the following result on the weak convergence of sequences in the Banach space of all continuous functions defined on a finite closed interval (see [7, 8]).

### Lemma B

$\lim_{n\rightarrow\infty}\int_{0}^{\pi}g_{n}\,dh_{x}=0$ *for all* $h_{x}\in BV[0,\pi]$ *if and only if* $\Vert g_{n} \Vert <\infty$ *for all* *n* *and* $\lim_{n\rightarrow\infty}g_{n}=0$.

We need the following well-known Dirichlet-Jordan criterion for Fourier series (see [8, 9]).

### Lemma C

*The trigonometric Fourier series of a* 2*π*-*periodic function* *f* *of bounded variation converges to* $[f(x+0)-f(x-0)]/2$ *for every* *x*, *and this convergence is uniform on every closed interval on which* *f* *is continuous*.

## Main results

We write

$$ \phi_{x}(t)=f(x+t)+f(x-t) $$

and

$$ h_{x}(t)=\frac{\phi_{x}(t)}{4\sin\frac{t}{2}}. $$

### Theorem 3.1

*Let* $f(x)$ *be a periodic function with period* 2*π* *and Lebesgue*-*integrable over* $[-\pi,\pi]$, *and let* $\tilde{S}_{k}^{\prime}(x)$ *denote the partial sums of series* (1.4). *Let the method* $\mathfrak{B}$ *be regular*. *Then*, *for every* $x\in{}[-\pi,\pi]$ *for which* $h_{x}(t)$ *is continuous and of bounded variation on* $[0,\pi]$,

3.1 $$ \lim_{n}\sum_{k}b_{nk}(i) \tilde{S}_{k}^{\prime}(x)=\frac{1}{4\pi}\int_{0}^{\pi}\operatorname{cosec}^{2} \frac{t}{2}\phi _{x}(t)\,dt-h_{x}(0+) $$

*uniformly in* *i* *if and only if*

3.2 $$ \lim_{n}\sum_{k}b_{nk}(i) \cos\biggl(k+\frac{1}{2}\biggr)t=0\quad\textit{for every }t\in {}[0, \pi]. $$

### Proof

We have

3.3 $$\begin{aligned} \tilde{S}_{k}^{\prime}(x) ={}&\frac{1}{\pi} \int_{-\pi}^{\pi}f(u)\frac {\partial}{\partial x} \Biggl( \sum _{p=1}^{k}\sin p(u-x)\,du \Biggr) \\ ={}&-\frac{1}{\pi} \int_{0}^{\pi}\frac{d}{dt} \biggl( \frac{\cos\frac {1}{2}t-\cos(k+\frac{1}{2})t}{2\sin\frac{1}{2}t} \biggr) \bigl( f(x+t)+f(x-t) \bigr) \,dt \\ ={}&-\frac{1}{\pi} \int_{0}^{\pi}\frac{d}{dt} \biggl( \frac{1}{2}\cos \frac{1}{2}t \biggr) \phi_{x}(t)\,dt+ \frac{1}{\pi} \int_{0}^{\pi}\frac{d}{dt} \biggl( \frac{\cos(k+\frac{1}{2})t}{2\sin\frac{1}{2}t} \biggr) \phi_{x}(t)\,dt \\ ={}&\frac{1}{4\pi} \int_{0}^{\pi}\operatorname{cosec}^{2} \frac {t}{2}\phi_{x}(t)\,dt-\frac{2}{\pi} \int_{0}^{\pi}\biggl(k+\frac{1}{2}\biggr)\sin \biggl(k+\frac{1}{2}\biggr)t\text{ }h_{x}(t)\,dt \\ &{}-\frac{1}{\pi} \int_{0}^{\pi}\frac{\cos(k+\frac{1}{2})t\text{ }}{\tan \frac{1}{2}t}h_{x}(t)\,dt \\ ={}&\frac{1}{4\pi} \int_{0}^{\pi}\operatorname{cosec}^{2} \frac {t}{2}\phi_{x}(t)\,dt+\frac{2}{\pi} \int_{0}^{\pi}\cos\biggl(k+\frac{1}{2}\biggr)t \text{ }\,dh_{x}(t) \\ &{}-\frac{1}{\pi} \int_{0}^{\pi}\frac{\cos(k+\frac{1}{2})t\text{ }}{\tan \frac{1}{2}t}h_{x}(t)\,dt \\ ={}&\frac{1}{4\pi} \int_{0}^{\pi}\operatorname{cosec}^{2} \frac {t}{2}\phi_{x}(t)\,dt+\frac{2}{\pi} \int_{0}^{\pi}\cos\biggl(k+\frac{1}{2}\biggr)t \text{ }\,dh_{x}(t)-M_{k}, \\ \tilde{S}_{k}^{\prime}(x)={}&\frac{1}{4\pi} \int_{0}^{\pi}\operatorname {cosec}^{2}\frac{t}{2}\phi_{x}(t)\,dt+\frac{2}{\pi} \int_{0}^{\pi}\cos\biggl(k+\frac {1}{2} \biggr)t\text{ }\,dh_{x}(t)-M_{k}, \end{aligned}$$

where

$$ M_{k}=\frac{1}{\pi} \int_{0}^{\pi}\frac{\cos(k+\frac{1}{2})t\text{ }}{\tan \frac{1}{2}t}h_{x}(t)\,dt, $$

and

3.4 $$\begin{aligned} \sum_{k}b_{nk}(i)\tilde{S}_{k}^{\prime}(x)={}& \sum_{k}b_{nk}(i)\frac {1}{4\pi}\int_{0}^{\pi}\operatorname{cosec}^{2} \frac{t}{2}\phi_{x}(t)\,dt \\ &{}+\frac {2}{\pi}\int_{0}^{\pi}D_{n}(t)\text{ }\,dh_{x}(t)-\sum_{k}b_{nk}(i)M_{k} \end{aligned}$$

with

3.5 $$ D_{n}(t)=\sum_{k}b_{nk}(i)\cos \biggl(k+\frac{1}{2}\biggr)t. $$

Since $h_{x}(t)$ is of bounded variation on $[0,\pi]$ and tends to $h_{x}(0+)$, $h_{x}(t)\cos\frac{t}{2}$ has the same properties. Therefore by Lemma C we have

3.6 $$ M_{k}(t)\rightarrow h_{x}(0+)\quad\text{as }n\rightarrow\infty. $$

Since $\mathfrak{B}$ is regular, by Theorem A we have

3.7 $$ \lim_{n}\sum_{k}b_{nk}(i)M_{k}(t)=h_{x}(0+) $$

and

$$ \lim_{n}\sum_{k}b_{nk}(i) \frac{1}{4\pi} \int_{0}^{\pi}\operatorname {cosec}^{2} \frac{t}{2}\phi_{x}(t)\,dt=\frac{1}{4\pi} \int_{0}^{\pi}\operatorname {cosec}^{2} \frac{t}{2}\phi_{x}(t)\,dt. $$

Next, we have to show that (3.2) holds if and only if

3.8 $$ \lim_{n} \int_{0}^{\pi}D_{n}(t)\text{ }\,dh_{x}(t)=0. $$

By Lemma B we have that (3.8) holds if and only if

3.9 $$ \bigl| D_{n}(t)\bigr|\leq K\quad\text{for all }n\text{ and }t\in{}[0,\pi ] $$

and (3.2) holds, where *K* is a positive constant.

Since condition (3.9) is satisfied by condition (i) of Theorem A, it follows that (3.8) holds if and only if (3.2) holds.

This completes the proof of the theorem. □

### Theorem 3.2

*Let* $f(x)$ *be a periodic function with period* 2*π* *and Lebesgue*-*integrable over* $[-\pi,\pi]$. *Then*, *for every* $x\in{}[-\pi,\pi]$ *for which* $h_{x}(t)$ *is continuous and of bounded variation on* $[0,\pi]$, $(\tilde{S}_{k}^{\prime}(x))$ *is* $(E,q)$-*summable to* $\frac{1}{4\pi}\int_{0}^{\pi}$ *cosec* $^{2}\frac{t}{2}\phi _{x}(t)\,dt-h_{x}(0+)$ *if and only if* $(\cos(k+\frac{1}{2})t)_{k}$ *is* $(E,q)$-*summable to* 0 *for every* $t\in{}[0,\pi]$.

### Proof

It follows easily by choosing $i=0$ and $b_{nk}(i)=b_{nk}$ with

$$ b_{nk}= \textstyle\begin{cases} \frac{1}{(1+q)^{n}}\binom{n}{k}q^{n-k}, & 0\leq k\leq n,\text{ }q>0, \\ 0 & \text{otherwise.}\end{cases} $$

 □

### Theorem 3.3

*Let* $f(x)$ *be a periodic function with period* 2*π* *and Lebesgue*-*integrable over* $[-\pi,\pi]$. *Then*, *for every* $x\in{}[-\pi,\pi]$ *for which* $h_{x}(t)$ *is continuous and of bounded variation on* $[0,\pi]$, $(\tilde{S}_{k}^{\prime}(x))$ *is* $(E,q)(\bar{N},p_{n})$-*summable to* $\frac{1}{4\pi}\int_{0}^{\pi }\operatorname{cosec}^{2}\frac{t}{2}\phi_{x}(t)\,dt-h_{x}(0+)$ *if and only if* $(\cos(k+\frac{1}{2})t)_{k}$ *is* $(E,q)(\bar{N},p_{n})$-*summable to* 0 *for every* $t\in{}[0,\pi]$.

### Proof

It follows easily by choosing $i=0$ and $b_{nk}(i)=c_{nk}$ with

$$ c_{nk}= \textstyle\begin{cases} \binom{n}{k}q^{n-k}\frac{1}{P_{k}}\sum_{\upsilon=0}^{k}p_{\upsilon}, & 0\leq k\leq n,\text{ }q>0, \\ 0 & \text{otherwise.}\end{cases} $$

 □

## Conclusion

In this paper, we have characterized the $F_{\mathfrak{B}}$-convergence of conjugate derived Fourier series. We deduced special cases to obtain necessary and sufficient conditions for $(E,q)$-summability and $(E,q)(\bar{N},p_{n})$-summability of the conjugate derived Fourier series.

## References

[1] Bell HT (1973). Order summability and almost convergence. Proc. Am. Math. Soc..

[2] Steiglitz M (1973). Eine Verallgemeinerung des Begriffs der Fastkonvergenz. Math. Jpn..

[3] Lorentz GG (1948). A contribution to the theory of divergent sequences. Acta Math..

[4] Maddox IJ (1988). Elements of Functional Analysis.

[5] Hardy GH (1949). Divergent Series.

[6] Mishra VN, Khatri K, Mishra LN (2012). Approximation of functions belonging to $\operatorname{Lip}(\xi(t),r)$ class by $(N,p_{n})(E,q)$ summability of conjugate series of Fourier series. J. Inequal. Appl..

[7] Banach S (1932). Théorie des Operations Linéaires.

[8] Mursaleen M (2014). Applied Summability Methods.

[9] Zygmund A (1959). Trigonometric Series.

